# The Possibility and Cause of Relapse After Previously Recovering From COVID-19: A Systematic Review

**DOI:** 10.7759/cureus.10264

**Published:** 2020-09-05

**Authors:** Sarah M Elsayed, Mithun K Reddy, Pooja M Murthy, Ishita Gupta, Monika Valiuskyte, Diana F Sánchez, Mark Anthony Diaz

**Affiliations:** 1 Internal Medicine, Ain Shams University, Faculty of Medicine, Cairo, EGY; 2 Medicine, Vydehi Institute of Medical Sciences and Research Centre, Bangalore, IND; 3 Medicine, Dr. Rajendra Prasad Government Medical College, Tanda, IND; 4 Family Medicine, Lithuanian University of Health Sciences, Kaunas, LTU; 5 Medicine, Cayetano Heredia Peruvian University, Lima, PER; 6 Infectious Disease Medicine, Avera Medical Group, Sioux Falls, USA

**Keywords:** relapse covid, corona relapse, covid-19 relapse, recurrence covid-19

## Abstract

The severe acute respiratory distress syndrome coronavirus-2 (SARS-Cov-2) is a novel coronavirus that is believed to be mainly transmitted via droplet and contact transmission. While research is focusing on epidemiology, transmission, vaccine development, and therapeutics for coronavirus disease 2019 (COVID-19), there is a possibility of disease relapse. There are reports of patients who tested positive for SARS-Cov-2 after clinical recovery and initial clearance of the virus.

Objective

This systematic review aims to identify the trends of COVID-19 relapse, the effects of co-morbidities on it, and associated mortality rates.

Methods

We conducted a systematic search during March and April 2020 for research articles on the relapse of COVID-19 using two primary databases, PubMed and Embase.

Results

A total of 13 eligible studies were screened of which 11 (case reports) were eligible for data extraction. The earliest to report relapse was after two days of discharge and the latest was 22 days after discharge. The mean number of days to relapse was 12 days and the median number was seven days. There was incomplete information about comorbidities. No mortalities were reported at the time of the study.

## Introduction

SARS-Cov-2 is a novel coronavirus that was first identified in Wuhan, China, at the end of 2019. This virus causes a respiratory illness named COVID-19 (referring to the year 2019) and has grown to be a global pandemic as declared by the World Health Organization (WHO) in March 2020. Currently, the virus is believed to be transmitted via droplets and contact transmission although speculations about airborne transmission exist [1]. While research is focusing on the epidemiology, transmission, vaccine development, and therapeutics for COVID-19, there remain gaps in our understanding of the natural history of this disease. One of those gaps is the possibility of disease relapse. There have been reports of patients who tested positive for SARS-Cov-2 after clinical recovery and initial documented clearance of the virus. Multiple explanations could exist, including disease relapse or reinfection. Being able to identify and accurately define disease relapse is of utmost importance, as this will allow researchers to recognize whether reinfection exists, which would have major implications on efforts to prevent infection. Recurrence or recrudescence refers to the reappearance of symptoms in survivors due to the persistence of the virus at immunologically segregated body sites [2]. Reinfection refers to survivors being susceptible to acquiring new infections after recovery. Patients reinfected with a strain determined to be of a different genotype or subtype than the previous strain they were infected with can easily be identified using genotyping assays. However, when reinfection with a similar strain of the same subtype occurs, phylogenetic analysis is required to distinguish reinfection from a virologic relapse [3]. Disease relapse has seldom been reported and there is no current consensus on its definition. In the quest to better understand the natural history of COVID-19, this systematic review of published evidence aims to identify the trends of COVID-19 relapse, the effects of co-morbidities on its relapse incidence, and relapse-associated mortality rates.

## Materials and methods

Search method and strategy

We conducted a systematic search during March and April 2020 for research articles on the relapse of COVID-19. Two primary databases were used, PubMed and Embase. The search strategy used the keywords COVID-relapse, relapse COVID, recurrence COVID 19, and corona relapse and was comprehensive with the cross-checking of reference lists from the articles retrieved. The Preferred Reporting Items for Systematic Reviews and Meta-Analysis (PRISMA) guidelines were used. This study is registered with the PROSPERO protocol (registration: CRD42020178476).

Data screening and eligibility

**Figure 1:**
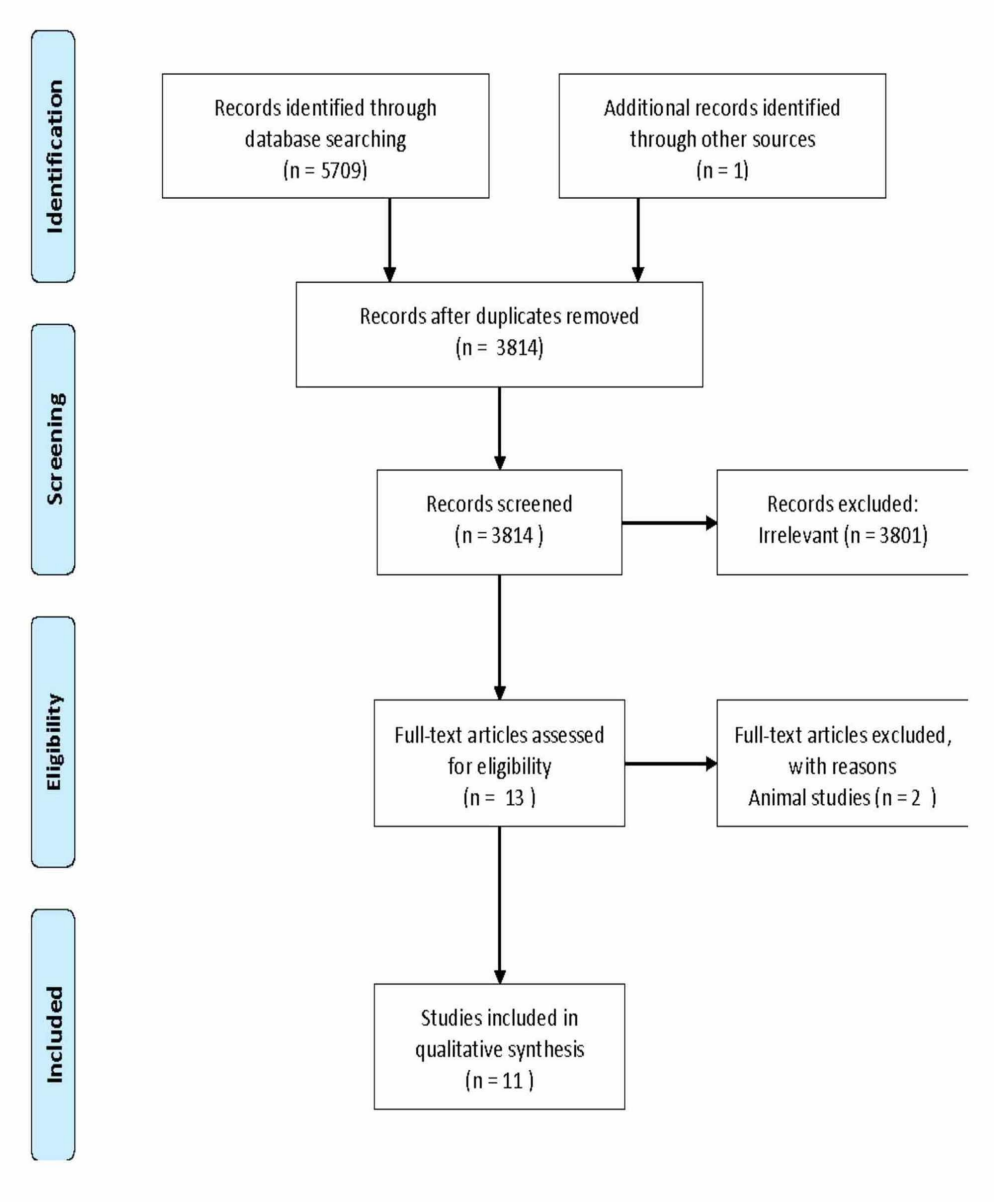
Preferred Reporting Items for Systematic Reviews and Meta-Analyses: The PRISMA statement data collection and analysis

We identified 5,709 abstracts via a literature search, of which 13 full-text articles were reviewed (Figure 1 ). Of these, 11 publications met the inclusion criteria for the final evaluation. A total of 3801 articles were excluded, as they were not relevant to the aim of our study and did not contain any information on the relapse of COVID-19. The publications included COVID-19 positive patient data and the relapse of disease was confirmed by PCR; the full text was available for these publications. Articles that included lab studies were excluded. Each publication was reviewed by two reviewers independently, and disagreements were discussed amongst all reviewers and resolved via consensus. Figure 1 illustrates the Preferred Reporting Items for Systematic Reviews and Meta-Analyses: The PRISMA Statement [4].

Data collection and analysis

Data were collected in the following categories when available: Study design; Study country; Patient demographics; Clinical signs and symptoms; Laboratory findings; Imaging studies; Dynamics of the oropharyngeal swab test; Treatment of the first presentation; The clinical picture of relapse; Day of a positive result after confirmed negative

We tabulated the data using Microsoft Excel (2010, Microsoft Corp, Redmond, WA). The data that were included in these tables were checked for accuracy by all authors. Statistical analysis was done by authors. This study did not require institutional review board (IRB) approval as data was obtained from already available databases, and patients were not directly involved.

Risk of bias assessment

Two authors independently assessed the risk of bias of each study included. The authors resolved disagreements by consensus, and a third author was consulted to resolve disagreements if necessary.

## Results

A total of 13 eligible studies were screened, of which 11 (case reports) were eligible for data extraction. The study reports a total of 11 patients (6 females and 5 males), all from China, who tested positive for COVID-19. All of them met the symptoms and testing criteria for discharge, which are: (i) Afebrile for at least three consecutive days, (ii) Improvement in respiratory symptoms, (iii) Improvement in chest imaging studies, computed tomography (CT), or X-ray, and (iv) Two negative reverse transcription-polymerase chain reaction (PCR-RT) tests 24 hours apart [5]. All of them were sent for precautionary quarantine for 14 days, as knowledge about the nature of the virus was not established yet. There is no information about home disinfection before discharge. The 11 cases reported a relapse, the earliest report was after two days of discharge and the latest was 22 days after discharge. The mean number of days to relapse was 12 days and the median number was seven days. The patients were mostly females and less than 40 years old. Six patients presented with symptoms (54.54%) and five were asymptomatic (45.45%). The most common symptoms with which relapsed patients presented were fever and fatigue (36.4%).

## Discussion

Coronaviruses are enveloped ribonucleic acid (RNA) viruses that can cause multiple system disorders in the human body. Six types of coronaviruses are known to infect humans. Two of them cause acute respiratory distress syndrome (ARDS) - the Severe Acute Respiratory Syndrome Coronavirus (SARS-CoV), which caused an outbreak in 2002 in China, and the Middle East Respiratory Syndrome Coronavirus (MERS-CoV) that caused an outbreak in the Middle east in 2012. The novel SARS-CoV2 is a betacoronavirus that also causes ARDS and can be transmitted between humans. SARS-CoV2 uses the angiotensin-converting enzyme (ACE) 2 receptor as a receptor for cell invasion. This is a similar mechanism to SARS-COV [6].

The typical presentation of SARS-CoV2 infection is fever, dry cough, dyspnea, fatigue, and lymphopenia [6]. In late April, the Centers for Disease Control and Prevention (CDC) announced that a new loss of taste or smell, headache, muscle pain, sore throat, and repeated shaking with chills can be associated with SARS-CoV2. It might result in severe acute respiratory syndrome (SARS) and even death in severe cases [6]. The general population is susceptible to SARS-CoV-2 as the main ways of transmission are respiratory droplets and contact. SARS-CoV-2 infected individuals are the primary source of infection in humans even if they are asymptomatic carriers [7]. Many patients have associated comorbidities [8].

In China, by the end of February, 39,002 patients were discharged from hospitals after meeting the set discharge criteria. Fourteen percent of the discharged patients showed positive nucleic acid re-examination or regained fever within one week [9]. In this study, we reviewed published reports of such patients in an attempt to better understand this phenomenon.

After a comprehensive review of the available literature, we identified 11 adult patients, all reported in China, who tested positive after being discharged and were thought to be recovering. Table 1 outlines the patients’ demographics and the initial clinical presentation. Of the 11 patients, five were males (45.5%) and six were females (54.5%). Six (54.5%) were less than 40 years old and five (45.5%) were older than 40 years old, including one patient older than 55 years old (9.1%). One patient (9.1%) had a past medical history of resolved tuberculosis, four (36.4%) had no comorbidities such as hypertension, diabetes, kidney disease, liver disease, cardiac disease, or immunosuppression. There is no data about the comorbidities of the remaining six patients (54.5%). Nine of the patients had an initial presentation with fever (81.8%) and eight had shortness of breath (72.7%). All of them were RT-PCR positive. Ten patients (90.9%) had CT findings of ground-glass opacities or lobar infiltrates at the time of presentation while one (9.1%) had no CT findings at all. All the patients were admitted for isolation and treatment in hospitals. All patients received empiric antiviral therapy either with the neuraminidase inhibitor oseltamivir or with the membrane infusion inhibitor Umifenovir along with an empiric antibacterial, most commonly a fluoroquinolone.

**Table 1:** Demographics of the patients and data about the first attack

Item	N
Gender	
● Male	5 (45.5%)
● Female	6 (54.5%)
Age	
● Less than 40 years old	6 (54.5%)
● More than 40 years old	5 (45.5%)
The clinical picture of the first presentation	9 (81.8%)
● Fever	3 (27.3%)
● Cough	1 (9.1%)
● Myalgia	1 (9.1%)
● Shortness of breath	8 (72.7%)
● Fatigue	1 (9.1%)
● Headache	1 (9.1%)
● Pharyngalgia	
Nucleic acid +ve oropharyngeal swabs at first attack (RT-PCR)	11 (100%)
CT findings	10(90.9%)
● Yes	1 (9.1%)
● No	
Treatment received after first attack	11 (100%)
● Yes ( Oseltamivir or Arbidol + Moxifloxacin)	4 (36.4%)
o Corticosteroids included in treatment	7 (63.7%)
o No details about corticosteroids in treatment	
Discharge criteria: (afebrile for 3 consecutive days, improved respiratory symptoms, improved chest imaging studies, 2 negative RT- PCR 24 hours apart) [6]	
● Met	11 (100%)
● Not met	0
Reported Comorbidities.	4 (36.4%)
● None	
● Yes	1 (9.1%)
● Age more than 55 years old	0 (0%)
● Diabetes	0 (0%)
● Hypertension	0 (0%)
● Renal disease	0 ( 0%)
● Liver disease	0 (0%)
● Cardiac disease	0 (0%)
● Pulmonary disease	1(9.1%)
● Immunosuppression	6 (54.5%)
● Not reported	

While it was not clear whether seven (63.7%) patients received corticosteroids, four patients (36.4%) had corticosteroids included in their treatment. Patients met the severe COVID-19 pneumonia criteria when: (i) respiratory rate per min ≥ 30; (ii) oxygen saturation ≤ 93% at resting state; and (iii) arterial blood oxygen partial pressure/oxygen concentration ≤ 300 mmHg [10]. It is proven that corticosteroids are beneficial in the treatment of acute respiratory distress syndrome (ARDS). Also, it is believed to prevent the progression of severe COVID-19 pneumonia to ARDS by suppressing the pro-inflammatory response and the cytokine storms if administered at five to seven days of onset [10]. This resulted in earlier fever recovery (2 days vs. 5 days, P = 0.010) as well as quicker improvement in Spo2 (8 days vs. 13.5 days P = 0.001) [10].

One-hundred percent (100%) of these patients met the pre-set criteria for discharge (mentioned earlier) [5]. After discharge, all cases were kept under surveillance and quarantined at home for at least 14 days; all cases had an RT-PCR oropharyngeal swab test for SARS-CoV-2 every day or every other day, at least five times [11].

Table 2 outlines the clinical presentation of the relapse. Out of the 11 patients, five (45.5%) were asymptomatic when re-tested positive, five (45.5%) had a fever, five (45.5%) had fatigue, four (36.4%) had fatigue associated with the fever, two (18.9%) had a cough, and two (18.9%) had a sore throat. All the patients had positive RT-PCR tests at the time of relapse. These were oropharyngeal swab-based in nine patients (81.8%) and sputum-based tests in the remaining two (18.9%). To note, the two patients who had positive sputum had negative oropharyngeal swabs. Four (36.4%) patients presented with relapse within seven days, four (36.4%) presented after seven days, and three (27.3%) presented with relapse after 14 days. There was no mortality reported among those 11 patients. There was not enough data reported about medical treatment received at the time of relapse.

**Table 2:** Presentation at the time of relapse

Item	N
Symptoms:	
● Asymptomatic	
● Fever	5 (45.6%)
● Cough	5 (45.6%)
● Fatigue	2 (18.2%)
● Fatigue with fever	5 (45.6%)
● Sore throat	4 (36.4%)
	2 (18.2%)
Positive nucleic acid swab:	
● Oropharyngeal:	
● Sputum	9 (81.8%)
	2(18.2%)
Duration:	
● Less than 7 days	
● More than 7 days	4 (36.4%)
● More than 14 days	4 (36.4%)
	3 (27.3%)
● No reported death of the cases studied	
● No data about treatment received for the relapse	

This study highlights the possibility of COVID-19 relapse. The patients reported were mostly females and less than 40 years old. The common presentations of the relapse are asymptomatic presentation, fever, or fever associated with fatigue within 14 days of discharge, although 27.3% of cases reported after 14 days up to 22 days [7].

We could not provide a correlation between the severity of the first presentation or comorbidities with the relapse. Thirty-six point four percent (36.4%) of the patients received corticosteroids, which suggests a potential link with the relapse that requires further studies [9]. There is no data about hospital readmission or treatment received for the relapse. No mortalities were reported in the patients included in our study.

There are possible explanations that require further studies. As the most common comorbidity of COVID-19 patients, diabetes, and hypertension may affect the prognosis of the disease [8]. We need more data if this could affect the possibility of getting a relapse. Age, immune status, underlying lung disease, and the severity of the SARS-CoV2 infection could all affect the elimination of the virus [8]. Furthermore, the cell entry receptor for COVID-19 is the angiotensin-converting enzyme-2 (ACE-2) receptor, which is mainly located on type 2 pneumocytes rather than in the upper respiratory tract, which could occasionally lead to false-negative results for oropharyngeal or nasopharyngeal swab tests due to the lower viral load in these specimens [5,7,11].

So, an important question is whether individuals upon recovery are prone to repeat infection. But, unfortunately, until now, it is an unresolved question. Several studies in the last four decades have shown that infections with the four endemic coronaviruses (229E, OC43, NL63, and HKU) are common in the general population. Natural re-infections with the same virus type have been documented previously, in which repeated infections with OC43 and 229E were recorded by serological testing. Subsequent infections were separated by at least eight months, though study participants were tested every four months. In most cases, re-infection occurred, though it could present with mild symptoms and a shortened duration of shedding [12].

The development of immunity to a pathogen through natural infection is a multistep process that typically takes place over one to two weeks. The body responds to a viral infection immediately, with a non-specific innate response in which macrophages, neutrophils, and dendritic cells slow the progress of the virus and may even prevent it from causing symptoms. This non-specific response is followed by an adaptive response where the body makes antibodies that specifically bind to the virus. These antibodies are proteins called immunoglobulins. The body also makes T-cells that recognize and eliminate other cells infected with the virus. This is called cellular immunity. This combined adaptive response may clear the virus from the body, and if the response is strong enough, may prevent progression to severe illness or re-infection by the same virus. This process is often measured by the presence of antibodies in the blood [13-19]. Most of these studies show that people who have recovered from infection have antibodies to the virus [13-15].

Tests to detect antibody responses to COVID-19 in the population will be critical to support the development of vaccines and to add to our understanding of the extent of infection among people who are not identified through active case finding and surveillance efforts, the attack rate in the population, and the infection fatality rate. For clinical diagnosis, however, such tests have limited utility because they cannot quickly diagnose acute infection to inform actions needed to determine the course of treatment [20].

Fang et al. did a study in February 2020, which compared the sensitivity of chest CT with that of RT-PCR. Their case series had 51 patients and they found that the sensitivity of CT for COVID-19 infection was 98% as compared to RT-PCR sensitivity of 71% with P < .001. Their study showed that the CT scan was more sensitive for COVID-19 [21]. Considering this, it is possible that the RT-PCR tests that were negative in our patients on discharge could have been a false negative. The role of chest CT in the evaluation of possible relapses needs to be further explored.

New research is directed to develop effective neutralizing antibodies (nAbs) for COVID-19 to be used as prophylactic and therapeutic agents to treat its infection and control its spread. In vitro studies showed that a mix comprising antibodies specific for RBD and specific regions in the S protein from SARS-CoV can cross-react and neutralize those of SARS-CoV-2. This theoretically can improve the breadth and potency of nAbs against the virus. Also, human sera from recovered patients can be used to treat COVID-19, but still, studies are needed to make sure it is not a source of infection and to identify its effect on the immune response [22].

Our study has some limitations. These include the small sample size with all the reported patients being adults, incomplete reporting of comorbidities in the included studies in this systematic review, and the absence of reports about relapses outside China. In the end, it is not clear whether these included reports represent a true relapse, prolonged shedding of the virus, or represent false-negative results on discharge. The effect of immunosuppressive therapy given for severe cases on the potential for relapse needs to be further explored. Based on this, a broader and larger study is necessary to further investigate the causes of the relapse.

## Conclusions

According to a recent scientific brief by the World Health Organization (WHO), there is no evidence that the patients who recovered from COVID-19 are immune from a second attack. The exact reason for that requires further studies to determine if it is a relapse or reinfection of the virus has not been completely eradicated from the body. It is unclear if the immune system develops neutralizing antibodies against a COVID-19 infection and if corticosteroid administration hinders their development. Also, we need to further know if the individual in the second attack is as contagious as the first one even if they develop neutralizing antibodies. Until then, it should be assumed that recovered cases are still at risk of reinfection. Accordingly, infection prevention guidelines set by the CDC and WHO should remain in practice.
